# Challenges of clinical translation from single‐cell sequencing to measures in clinical biochemistry of haematology: Definition of immune cell identities

**DOI:** 10.1002/ctm2.1401

**Published:** 2023-09-12

**Authors:** Xuanqi Liu, Diane C. Wang, Charles A. Powell, Xiangdong Wang

**Affiliations:** ^1^ Department of Pulmonary and Critical Care Medicine Zhongshan Hospital Shanghai Engineering Research for AI Technology for Cardiopulmonary Diseases Fudan University Shanghai Medical College, Shanghai Institute of Clinical Bioinformatics Shanghai China; ^2^ Department of Emergency Medicine Princess Alexandra Hospital Brisbane Australia; ^3^ Division of Pulmonary Department of Critical Care and Sleep Medicine Icahn School of Medicine at Mount Sinai New York New York USA

**Keywords:** cell identity, clinical, medicine, RNA sequencing, single‐cell, translational

## Abstract

Peripheral immune cells play important roles in the maintenance of systemic and microenvironmental hemostasis. Measurements of circulating blood cells by single‐cell RNA sequencing (scRNA‐seq) were proposed as one of the routine measures in clinical biochemistry of hematology. Out of translational challenges, defining precise identities of cell subsets and states is more difficult, due to the complexity of immune cell development, location, regulation, function, and metabolism. It is also a challenge to precisely interpret clinical significance and impact of each cell identity marker gene panel (ciMGPs). ciMGPs have potential to advance the understanding of systemic responses of the disease, identify disease‐specific biomarkers, and to define cell heterogeneity. Recently, a large number of peripheral cell subsets and expending/activating states have been identified and validated for use in the fast developments in clinical single cell biomedicine. Defining specificity, measurability, and repeatability of cell subsets/states is important for translation of peripheral scRNA‐seq in clinical hematology and biochemistry. The development of standard operating procedure and performance of clinical trials in large populations at various ages, diseases, and therapies will promote the clinical translation of ciMGPs to measures. Thus, defining cell subset/state identities will provide the multi‐dimensional and comprehensive readouts of systemic immune cells, the precision monitoring of immune dynamics, and deeper‐understanding of the disease and response to therapy.

1

Systemic and local immune cells play decisive roles in the maintenance of microenvironmental hemostasis and the development of cancer, inflammation and ageing. Depending on the context, immune cells form a defence zone against or promote invasion and metastasis of cancer cells in the tumour microenvironment, processes that are highly dependent upon the nature of the disease, spatialization of immune cell subsets/subclusters and interventions.^1^ The microenvironment immune cells are associated with cancer metastases, response to therapies and prognosis of patients. The spatial heterogeneity, activation and complexity of immune cells and their communication with tissue structure cells are influenced by ageing and systemic inflammation.^2^ Changes in immune cell state, gene expression and spatial organization were more obvious in tissue structure cells.

The interplay between systemic and local immune cell dynamics and phenomes is dependent upon the disease characteristics, severities, and stages. For example, the single‐cell multi‐omic profiling of matched nasal, tracheal, bronchial and peripheral blood immune cells demonstrated a consistent response to interferon in pediatric patients with coronavirus disease 2019 infection.^3^ While, the consistency and matching between systemic peripheral and local microenvironment immune cell profiles can vary tremendously, due to the complexity that microenvironmental immune cells recruited from the circulation can infiltrate/be trapped into the tissue interstitial space and amongst cancer and tissue structure cells or still circulating within the capillaries. Measurements of circulating blood cells by single‐cell RNA sequencing (scRNA‐seq) are proposed for development as routine measures in clinical biochemistry of haematology for comprehensive indication of physiological and pathophysiological conditions and for better understanding of disease complexities.^4^ Clinical Single‐Cell Biomedicine is an important first step to success in the translation of single‐cell biology to clinical practice.^5^

scRNA‐seq cell identity marker gene panels (ciMGPs) are helpful in characterizing the ever‐increasing number of circulating immune cell subsets/subclusters. For example, the subset/subcluster number of neutrophils and monocytes categorized by ciMGPs are estimated to be more than 100, T‐lymphocytes about 300, B‐lymphocytes about 150, dendritic cells about 100 and nature killing cells about 70. One of the biggest challenges during the clinical translation is to select and optimize human‐ and disease‐specific immune cell subsets/clusters according to the specificity, measurability, and repeatability of ciMGPs. In order to define the specificity of ciMGPs, one needs to be able to trace sources, clarify the developmental trajectory, identify cell transition states or stable subsets/subclusters, and form evaluation criteria and standards. Of those, the regional overlap expression rate of ciMGPs in a cell subset/subcluster of lung tissue single cells was proposed as the benchmark to measure the specificity and accuracy of the ciMGPs.^6^ Cell types/subsets were categorized as cell‐specific, cell‐associated and cell‐reference, although it still remains unclear whether the overlap expression rate might be suitable for monitoring dynamically altered circulating immune cells, uncovering various functional transition states of subset cells, and differentiating the states and subsets/subclusters.

The complexity of immune cell development, location, regulation, function, and metabolism presents another challenge to the definition of cell subset identities. Multiple states of immune cells occur during differentiation, activation and response to extracellular stimuli to make the definition even more difficult and sophisticated, including functional and dysfunctional states, transition and stable states, hypo/hyperresponsiveness states, and immune‐paralysis states. For example, T‐cell exhaustion was defined as a T‐cell dysfunctional state and characterized by clear alterations of effector function, inhibitory receptor expression, and transcriptional state in many pathological conditions such as infections, severe diseases or cancer.^7^ Transcriptomic phenomes of immune cells can be dynamically reprogrammed from functional to dysfunctional cell states, during which ciMGPs‐based subsets/subclusters are concurrently reshaped. It is possible that multiple cell subsets/subclusters are flickering or sustained in a dysfunctional state, or one subset may persistently appear in many states, all of which may be dependent upon the nature and severity of the disease. Based on scRNA‐seq data, Zhang's team established a comprehensive atlas of T cells from 316 donors across 21 cancer types and evidenced that various states and formations of multiple potentially tumour‐reactive T cell populations/subsets and state‐transition paths depended upon the cancer cell types in the tumour microenvironment.^8^ This study demonstrated a strong state transition from TCF7‐positive exhausted T cells to GZMK‐positive exhausted T cells and to terminal exhausted T cells in the tumour microenvironment. In the Alzheimer's disease brain, the T‐cell phenomes and numbers were transformed from activated to exhausted states, accompanied by neuronal loss and the development of tauopathy.^9^ In addition to considering the complexity and diversity of immune cell phenomes and states, the ciMGP appearance in the circulation and local tissue can be a subset of immune cells, as long as measurable and repeatable.

As the number and accuracy of ciMGPs‐based subsets and locations are increasing with the rapid development of scRNA‐seq and spatial transcriptomes, it is also a challenge to precisely interpret the clinical significance and impact of each immune cell subset. There remains an incomplete understanding of what the ciMGPs expression represents, the functional value of those gene combinations, and the medical impact on disease diagnosis and therapy. The nomenclature of ciMGPs‐based subsets remains to be clarified and standardized since the current naming is diverse and confusing. There is an urgent need to have a global consensus and standardization on the identification, validation, reference values, methodology, and nomenclature of ciMGPs‐based immune cell subsets/subclusters in order to facilitate the development of new insights into understanding clinical impacts and applications. The success of ciMGPs‐based immune cell subset measures in the clinical biochemistry of haematology may become a milestone of spatiotemporal molecular imaging and medicine.^10^, ^11^ Spatialization and temporization of ciMGPs‐based subsets can be illustrated by integrating scRNA‐seq with the spatial transcriptome.^12^ It is important to define the correlations and trajectories between circulating and spatial immune cell subsets and to determine if circulating ciMGPs indirectly reflect the spatialization and state in the microenvironment. By integrating spatial single‐cell in situ technology with other methods,^13^ we expect that the molecular characterization and subcellular spatial resolution of circulating immune cell subsets can be clearly detected and used to better understand the complex and diverse network of cells within the microenvironment.

Single‐cell transcriptomic landscapes and alterations of peripheral blood immune single cells have become one of the most important approaches to understanding systemic responses of the disease, uncovering disease‐specific biomarkers, and exploring transcriptional and genetic heterogeneity. The methodologies of peripheral immune single‐cell isolation, purification, analysis, and annotation have improved over more than a decade, with a focus on special or rare cell types. During the clinical translation of peripheral scRNA‐seq, an alternative is to develop a cell‐type‐specific ciMGPs‐based approach for human peripheral blood. Rheumatoid arthritis‐specific cell populations, local heterogeneity of cell states, and corresponding inflammatory mediators with special cell populations were uncovered by integrating RNA sequencing and protein detections.^14^ The single‐cell atlas of peripheral immune cell phenotypes was mapped in pregnant women spanning the entire gestation period and proposed to predict the gestational age of normal pregnancy by a cell‐type‐specific model.^15^ RNA/Protein‐based phenomes and specificities of peripheral immune cell subsets or states will be well described and characterized with multi‐measures and correlation analyses, while biological functions and responses of those cells remain to be deeply explored. Reyes et al found that peripheral blood cell states were significantly correlated with the septic status and identified a sepsis‐specific monocyte state MS1, one of the CD14+ cells with high expression of RETN, ALOX5AP and IL1R2, in the blood of sepsis patients.^16^ From a clinical translation point of view, a panel of surface proteins (low HLA‐DR and high IL1R2) was defined as a cytologic marker of the MS1 cell state, after being validated by comparison with previously reported classifiers and other RNA‐seq datasets. This particular study sets an example of translation from ciMGPs‐based subsets and states into clinically measurable protein‐based panels and defines the developmental source and molecular functions of selected target cell subsets.

As a new discipline of clinical single‐cell biomedicine, it will be important to clarify the cellular heterogeneity at single‐cell resolution and to overcome other obstacles on the pathway of clinical translation from scRNA‐seq into application. Analyses of scRNA‐seq data and the establishment of databases with self‐learning capacity are critical to improving the quality and quantity of clinical reports. The role of bioinformatics for clustering cell subsets and states, clinical bioinformatics for integrating clinical phenomes with single‐cell transcriptomic profiles, and trans‐omics for multi‐dimensional understanding is paramount. In order to meet clinical requirements of precisely efficient processing, measuring, analyzing, and reporting, there are urgent need to optimize performances, standardize protocols, and create an artificial intelligence single‐cell model as proposed previously.^17^ In addition to the general burdens of scRNA‐seq such as high costs, complex operations, and equipped systems, the peripheral blood scRNA‐seq has its own challenges such as repeatable sampling, resolution of clear components, and accurate assessments of microenvironmental immunity.

In conclusion, there is increasing evidence that a large number of circulating immune cell subsets and expanding/activating states are being identified and validated with the rapid development of clinical single‐cell biomedicine. The specificity, measurability, and repeatability of cell subsets/states are important for the translation of peripheral scRNA‐seq in clinical haematology and biochemistry. Based on the knowledge from rapidly developing peripheral scRNA‐seq methodologies and ciMGPs, we are expecting standard operating procedures and clinical trials with large populations of various ages, diseases, and therapies. With ciMGPs optimization of peripheral cell subsets/states, we are expecting multi‐dimensional and comprehensive readouts of systemic immune cells to facilitate precision monitoring of immune dynamics, and a deep understanding of the disease and response to therapy.

## CONFLICT OF INTEREST STATEMENT

The authors declare no conflict of interest.

## FUNDING INFORMATION

Not applicable.

2

## Data Availability

Data sharing is not applicable to this article as no new data were created or analyzed in this study.
